# Reproducibility of 18F-fluoromisonidazole intratumour distribution in non-small cell lung cancer; methodological issues to avoid mismanagement of the patients

**DOI:** 10.1186/s13550-017-0270-7

**Published:** 2017-03-16

**Authors:** Siamak Sabour

**Affiliations:** 1 Safety Promotion and Injury Prevention Research Center, Shahid Beheshti University of Medical Sciences, Tehran, I.R. Iran; 2 Department of Clinical Epidemiology, Shahid Beheshti University of Medical Sciences, Tehran, I.R. Iran

**Keywords:** Reproducibility, FMISO, PET, NSCLC, Methodology, Mismanagement

I was interested to read the paper by Grkovski M and colleagues published in the Dec 2016 issue of EJNMMI Res [1]. They aimed to assess the reproducibility of 18F-fluoromisonidazole (FMISO) positron emission tomography (PET) as a non-invasive, quantitative imaging technique, spatiotemporal intratumour distribution in patients with non-small cell lung cancer (NSCLC) [1]. The Pearson correlation coefficient *r* was calculated for mean standardized uptake values (SUV) within investigated volumes of interest and for voxels within tumour volumes (*r*_TV_). The reproducibility of FMISO voxelwise distribution, SUV- and tumour-to-blood ratio (TBR)-derived indices was assessed using correlation and Bland-Altman analyses [1]. Although they correctly used Bland-Altman, they reported Pearson’s correlation *r* which in reproducibility (precision, repeatability, reliability, or interchangeability) is one of the common mistakes [2–6]. Pearson’s correlation *r* only assesses the linearity between two continuous variables. Any shift in the location and/or scale of the regression line which leads to non-reproducibility cannot be detected by this correlation coefficient [2–6]. Therefore, for quantitative variables, Intra Class Correlation Coefficient single measure is the best statistical test to evaluate reproducibility [2–6].

Based on their results, the SUV_max_, SUV_mean_, TBR_max_, and TBR_mean_ were highly correlated (*r* ≥ 0.87, *p* < 0.001) and were reproducible to within 10–15% [1]. It is good to know that in reliability analysis, individual based approach should be considered instead of global average which Pearson’s correlation *r* cannot do. It means we can simply get strongly positive and significant Pearson *r* (*r* = 0.95, *p* value < 0.001) with no reproducibility at all. Moreover, statistically significant should not be considered in reproducibility analysis [2–6]. They concluded high reproducibility of FMISO intratumour distribution in NSCLC patients, facilitating its use in determining the topology of the hypoxic tumour sub-volumes for dose escalation, in patient stratification strategies for hypoxia-targeted therapies, and in monitoring response to therapeutic interventions. Such conclusion may be a misleading message due to inappropriate use of statistical test to assess reproducibility. Briefly, for reliability analysis, appropriate tests should be applied; otherwise, misdiagnosis and mismanagement of the patients cannot be avoided.
